# Construction of a Five-Super-Enhancer-Associated-Genes Prognostic Model for Osteosarcoma Patients

**DOI:** 10.3389/fcell.2020.598660

**Published:** 2020-10-30

**Authors:** Zhanbo Ouyang, Guohua Li, Haihong Zhu, Jiaojiao Wang, Tingting Qi, Qiang Qu, Chao Tu, Jian Qu, Qiong Lu

**Affiliations:** ^1^Department of Pharmacy, The Second Xiangya Hospital, Central South University; Institute of Clinical Pharmacy, Central South University, Changsha, China; ^2^Department of Pharmacy, Xiangya Hospital, Central South University, Changsha, China; ^3^Department of Orthopaedics, The Second Xiangya Hospital, Central South University, Changsha, China

**Keywords:** osteosarcoma, super-enhancer, prognostic model, Lasso, weighted gene co-expression network analysis, overall survival

## Abstract

Osteosarcoma is a malignant tumor most commonly arising in children and adolescents and associated with poor prognosis. In recent years, some prognostic models have been constructed to assist clinicians in the treatment of osteosarcoma. However, the prognosis and treatment of patients with osteosarcoma remain unsatisfactory. Notably, super-enhancer (SE)-associated genes strongly promote the progression of osteosarcoma. In the present study, we constructed a novel effective prognostic model using SE-associated genes from osteosarcoma. Five SE-associated genes were initially screened through the least absolute shrinkage and selection operator (Lasso) penalized Cox regression, as well as univariate and multivariate Cox regression analyses. Meanwhile, a risk score model was constructed using the expression of these five genes. The excellent performance of the five-SE-associated-gene-based prognostic model was determined via time-dependent receiver operating characteristic (ROC) curves and Kaplan–Meier curves. Inferior outcome of overall survival (OS) was predicted in the high-risk group. A nomogram based on the polygenic risk score model was further established to validate the performance of the prognostic model. It showed that our prognostic model performed outstandingly in predicting 1-, 3-, and 5-year OS of patients with osteosarcoma. Meanwhile, these five genes also belonged to the hub genes associated with survival and necrosis of osteosarcoma according to the result of weighted gene co-expression network analysis based on the dataset of GSE39058. Therefore, we believe that the five-SE-associated-gene-based prognostic model established in this study can accurately predict the prognosis of patients with osteosarcoma and effectively assist clinicians in treating osteosarcoma in the future.

## Introduction

Osteosarcoma, the most common primary bone tumor, is most commonly arising in children and adolescents (Bielack et al., 2009). The survival rate of patients with osteosarcoma is markedly low due to the tendency of osteosarcoma for early metastasis and rapid progression (Kempf-Bielack et al., 2005). The overall 5-year survival rate of patients with non-metastatic osteosarcoma is approximately 60–70%, while the survival rate of osteosarcoma patients with metastatic symptoms is only 20–30% (Kempf-Bielack et al., 2005; Luetke et al., 2014; Meazza and Scanagatta, 2016). Moreover, high-grade patients comprise nearly 80–90% of those diagnosed with osteosarcoma; the treatment of these patients is challenging (Bielack et al., 2009). Furthermore, despite advancements in treatment in recent years, the therapeutic outcomes remain unsatisfactory. Although numerous traditional predictive factors, such as age, necrosis, recurrence, and clinical stage, are important contributors to clinical outcome, they are less effective in predicting the survival status due to the complex molecular mechanisms of osteosarcoma progression. Thus, it is urgent to investigate novel effective molecular biomarkers for the more precise prediction of the prognosis of patients with osteosarcoma.

Super-enhancer is a large cluster of *cis*-regulatory DNA elements densely bound by transcription factors and cofactors, playing vital roles in defining cell fate and identity (Hnisz et al., 2013). Histone marks (H3K27ac, H3K4me1, and H3K4me3), cofactor (p300), and mediators (CDK7 and BRD4) are common biomarkers used to define SE (Lovén et al., 2013; Hnisz et al., 2015; Ma et al., 2019); among them, H3K27ac is the most efficient biomarker for identifying SE (Creyghton et al., 2010; Hnisz et al., 2013; Whyte et al., 2013). Therefore, we used H3K27ac, H3K4me1, and H3K4me3 as SE biomarkers in this study based on the ChIP–seq profiles data from Cistrome. Moreover, SE plays a critical role in the progression of osteosarcoma through activating the expression of its downstream target oncogenes, such as *MYC*, *LIF*, and *STAT3* (Chen et al., 2018; Lu et al., 2020; Zhang et al., 2020). Furthermore, SE inhibitors (e.g., JQ1, THZ1, and THZ2), which were developed for the treatment of osteosarcoma, could restore the expression levels of these genes (Lee et al., 2015; Chen et al., 2018; Zhang et al., 2020). Thus, screening and identifying hub oncogenes driven by SE will provide novel insights into the diagnosis, prognosis, and treatment of osteosarcoma.

The Lasso penalized Cox regression analysis, designed by Tibshirani (1997), is an accurate statistical method frequently used for the selection of variables in the Cox regression model. It can remarkably improve the accuracy of the model it generates compared with the stepwise regression method, as it involves fewer variables and produces more interpretable models (Tibshirani, 1997; Xiong et al., 2019). The reason for Lasso screening fewer variables is that it can regularize the impact of some variables by shrinking their coefficients to zero (Zhang et al., 2018). Nowadays, Lasso is widely used in constructing prognostic models for the prediction of survival based on complicated high-throughput genomic data (Zhang et al., 2013; Lu et al., 2019; Wang et al., 2019; Wu et al., 2019). Based on this advantage, we applied the Lasso regression method to the construction of a prognostic model of osteosarcoma.

Weighted gene co-expression network analysis (WGCNA) is a system bioinformatics method involving molecular interaction mechanism analysis and construction of correlation gene networks (Zhang and Horvath, 2005). WGCNA has been frequently utilized in identifying probable oncogenes and in screening candidate targets for various cancers (Udyavar et al., 2013). Using WGCNA, Tian et al. (2018) showed that the insulin-like growth factor-binding-associated genes may play a critical role in osteosarcoma metastasis progression. Guan et al. (2020) defined four key genes (*ALOX5AP*, *HLA-DMB*, *HLA-DRA*, and *SPINT2*) as prognostic markers for osteosarcoma metastasis. We adapted WGCNA to identify the five screened genes from SE-associated genes that were crucial hub genes of osteosarcoma. Prognostic models based on some cancer-related genes (Long et al., 2018; Zuo et al., 2019), long non-coding RNAs (Liu et al., 2020; Yang et al., 2020; Zhang et al., 2020), or other biomarkers (Park et al., 2017; Elwood et al., 2018) for improving patient prognosis attract considerable attention. For instance, Zhu et al. (2020) constructed a seven-gene signature associated with osteosarcoma energy metabolism for predicting the prognosis of osteosarcoma through WGCNA and Lasso Cox regression analysis. Dong et al. (2019) constructed a risk score model based on eight genes for predicting the metastasis of osteosarcoma mainly through Lasso logistic regression analysis. Thus, we attempted to perform WGCNA for osteosarcoma and screen hub genes related to clinical traits of this disease. Nomograms are interactive tools developed in recent years, which have been widely used to predict the probability of survival (Wang et al., 2018; Yap et al., 2018; Pan et al., 2019; Xiao et al., 2019; Zhang et al., 2019). Similarly, we constructed a prognostic model and a visualized nomogram based on this model to improve the prognosis of patients with osteosarcoma.

In this study, Lasso penalized Cox regression analysis was initially performed using 349 selected SE-associated genes. A gene cluster containing five SE-associated genes, namely, *AMN1*, *LIMS1*, *SAMD4A*, *SPARC*, and *ZP3*, was screened. Subsequently, a risk score model based on these five genes was constructed and verified using training and validation datasets. Meanwhile, univariate and multivariate Cox regression analyses and ROC curve analysis were also used to assess the predictive ability of the model. We further screened module genes related to clinical traits of patients with osteosarcoma via WGCNA. The five SE-associated genes were also part of the module genes linked to survival and recurrence of osteosarcoma. Finally, an interactive nomogram was constructed based on the risk score model including the five-gene risk group and clinical traits. The five-SE-associated-gene-based prognostic model developed in this study will provide promising inspiration for the clinical therapy of patients with osteosarcoma.

## Materials and Methods

### Data Collection and Expression Matrix Preparation

We downloaded the osteosarcoma dataset GSE39058 as training data from the National Center for Biotechnology Information GEO^1^. The 20,819 genes in 42 samples of osteosarcoma were annotated according to the probe information from Illumina HumanHT-12 WG-DASL V4.0 R2 Expression BeadChip (GPL14951) (Kelly et al., 2013). We processed the raw data of the expression matrix by transformation of log2– via the R package “limma.” The clinical information was organized into a matrix and is shown in Table 1. One sample (GSM954821) was excluded due to the lack of information on the survival status. The validation dataset Target-osteosarcoma (Target-OS) was downloaded from the publicly available database of TCGA^2^. The 434 SE-associated genes from the osteosarcoma cell line U2OS were obtained from the website of the SE database (version 1.03^3^). Following the intersection of SE-associated genes and 20,819 genes from GSE39058, 349 SE-associated genes were used for the subsequent analysis. Chromatin immunoprecipitation (ChIP) signals of H3K27ac-seq, H3K4me1-seq, and H3K4me3-seq in U2OS were also downloaded from Cistrome^4^ and visualized using the IGV (Thorvaldsdottir et al., 2013).

**TABLE 1:** The clinical information of 41 OS patients from dataset GSE39058.

Variable		Number
Gender	Male	21
	Female	20
Necrosis	Low	14
	High	27
Recurrence	No	21
	Yes	20
Death	No	29
	Yes	12

### Lasso Penalized Cox Proportional Hazard Regression Model

Lasso penalized Cox regression analysis was performed to screen important prognostic genes for predicting the OS of patients with osteosarcoma using R package “glmnet” (Friedman et al., 2010; Simon et al., 2011; Tibshirani et al., 2012). The shrinking penalty was identified based on the tuning parameter lambda (λ), which was identified based on 10-fold cross-validation. Two optimal λ values (λ_min_ and λ_lse_) were highlighted by the vertical lines with a minimizing mean-square error. To determine the optimal λ value, we performed the Wilcoxon test and ROC curve analysis for the comparison of λ_min_ and λ_lse_ values. These two λ values were used to construct a new fitted Lasso model, and two lists of genes were generated.

### Cox Proportional Hazard Regression Model

Univariate and multivariate Cox proportional hazard regression analyses were performed to validate the associations between the expression levels of selected genes and OS of the patients via the R package “survival” and “survminer.” The regression coefficient (β-value) and HR were predicted. Harrell’s C-index, likelihood ratio test, and Wald test were also calculated, and the results are presented in the forest plot. Survival analysis of the single gene in the prognostic model was performed using the K–M survival curve and log-rank test via the R package “survival” and “survminer.” Time-dependent ROC curve analysis was calculated to evaluate the predictive capability of OS of the prognostic model via the R package “timeROC.”

### Polygenic Risk Score Model for Prognostic Prediction

To further investigate the prognostic model based on the gene group, a polygenic risk score model was constructed used the following forum RiskScore = Σβi ^∗^ Xi. Xi is the level of gene expression and βi is the regression coefficient. The risk score model was defined as the linear combination of the expression levels of selected genes. The patients with osteosarcoma in the two datasets were divided into high- and low-risk groups according to the risk score. We verified the power of the polygenic risk score model through single-gene expression status analysis, event distribution analysis, K–M survival analysis, and time-dependent ROC curve analysis.

### Predictive Nomogram for Prognostic Prediction

A nomogram based on independent prognostic factors of clinical traits and the polygenic risk score was constructed to predict the probability of 1-, 3-, and 5-year OS of patients with osteosarcoma. Subsequently, the discrimination of the nomogram was verified using the C-index obtained through a bootstrap method with 1,000 resamples. Calibration curves were plotted to compare the nomogram-predicted and ideal probabilities against the observed rates. Total points were calculated according to the parameters of prognostic factors using the R package “nomogramEx.” Each variable of the nomogram yields points, and their sum represents the total points that a patient receives. Finally, we developed the interactive nomogram for predicting the probability of OS of patients with osteosarcoma using the R package “replot.”

### WGCNA

Weighted gene co-expression network analysis was performed to screen SE-associated hub genes linked to clinical traits using the dataset GSE39058. The analysis was performed as previously described (Ma et al., 2019). The optimal soft-thresholding value was estimated based on scale independence and mean connectivity analysis. Clinical traits-related genes were clustered into different modules. Moreover, the correlation between modules and clinical traits was highlighted in the heatmap. The relationship between the clinically significant MM and GS was displayed in the scatter plots.

### Protein–Protein Interaction Analysis and Pathway Enrichment Analysis

Eigengenes within clinically significant modules and SE-associated genes were intersected using the Venn plot and produced a list of hub genes. An interaction network between hub genes and drugs was also constructed to identify some drugs related with these hub genes via “NetworkAnalyst 3.0” (Zhou et al., 2019). Subsequently, Kyoto Encyclopedia of Genes and Genomes (KEGG) pathway analysis was performed using these hub genes. The top 10 items of the enriched pathway were selected and shown in the dot plot.

## Results

### Establishment and Validation of the Lasso Penalized Cox Regression Model

**FIGURE 1:**
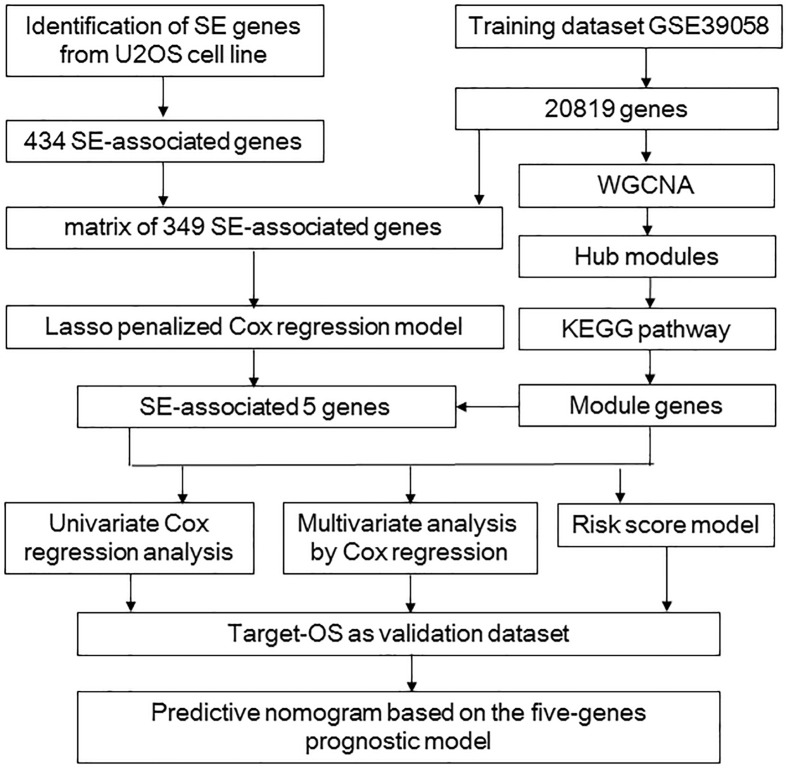
The procedure workflow used to establish and certify the SE-associated gene-based prognostic model for patients with osteosarcoma. KEGG, Kyoto Encyclopedia of Genes and Genomes; Lasso, least absolute shrinkage and selection operator; Target-OS, Target-osteosarcoma; SE, super-enhancer; WGCNA, weighted gene co-expression network analysis.

**FIGURE 2:**
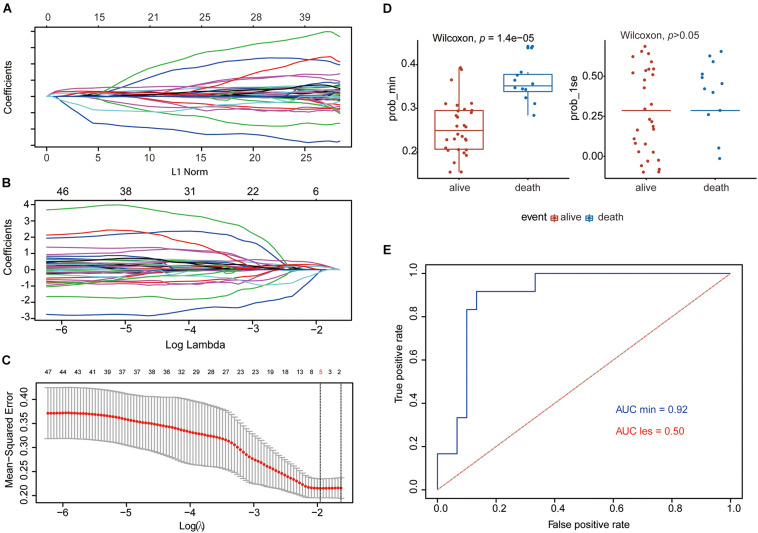
Establishment of the five-gene prognostic model by Lasso regression analysis based on the 349 SE-associated genes from data downloaded from SEdb. **(A**,**B)** Lasso coefficient profiles of the 349 SE-associated genes. **(C)** Two optimal lambda (λ) values (λ_min_ and λ_lse_) were estimated according to the vertical lines with a minimizing mean-square error. The vertical axis represents the mean-square error, while the horizontal axis represents the value of log(λ). **(D)** The scatter plot of survival status of patients with osteosarcoma based on the five-gene model (left, λ_min_, *p* = 1.4e−05) or two-gene model (right, λ_lse_, *p* > 0.05) using the Wilcoxon test. **(E)** ROC curves of the two prognostic models based on λ_min_ and λ_lse_. The values of AUC were included in the figure. AUC, area under the curve; Lasso, least absolute shrinkage and selection operator; ROC, receiver operating characteristic; SE, super-enhancer; SEdb, super-enhancer database.

The flowchart shows our analysis procedures for the construction of the SE-associated gene-based prognostic model of osteosarcoma (Figure 1). The 349 SE-associated genes based on the dataset of GSE39058 were selected to perform the Lasso penalized Cox regression analysis. Firstly, to identify the best-fit parameter of the λ parameter, two important optional λ values (logλ_min_ = −1.91 and logλ_lse_ = −1.48) were calculated from the vertical lines with a minimizing mean-square error and further used to select two groups of genes (Figures 2A–C). Moreover, Lasso models were reconstructed according to the λ_min_ and λ_lse_, and survival probabilities were further estimated based on two gene lists (Figure 2D). As shown in Figure 2D, the use of the five gene-based prognostic model based on the λ_min_ (Wilcoxon test, *p* = 1.4e−05) facilitated the obvious distinction of survival probabilities between samples obtained from living and expired patients. However, when a two-gene-based prognostic model was used based on the λ_lse_, there was no statistically significant difference observed (Wilcoxon test, *p* > 0.05). Similarly, the outcome of ROC curve analysis showed that the area under the curve minimum (AUC_min_) was 0.92, implying that the five-gene-based Lasso model performed well in predicting the probability of OS of patients with osteosarcoma (Figure 2E).

### Validation of Independent Prognostic Factors by the Cox Regression Model

**FIGURE 3:**
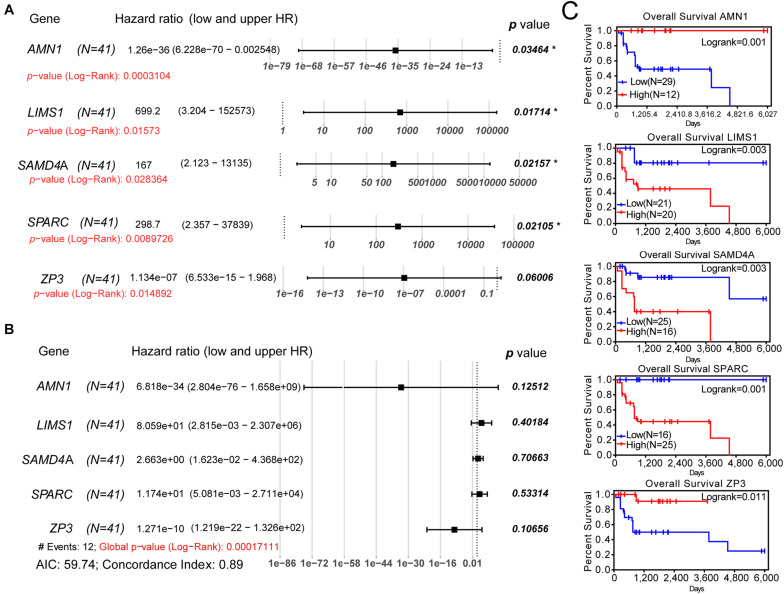
Univariate and multivariate Cox regression analyses and K–M survival analysis of the five-gene prognostic model. **(A)** Univariate Cox regression analysis of each gene of the model. **(B)** Multivariate Cox regression analysis of the five-gene model. **(C)** K–M survival curves showing the difference in OS between the relative high- and low-expression groups for each gene according to the median of expression levels. K–M, Kaplan–Meier; AIC, Akaike information criterion; OS, overall survival.

For the validation of the Lasso penalized Cox regression model, we performed univariate and multivariate Cox regression analyses to determine whether these genes were independent prognostic factors for the OS of patients with osteosarcoma. In the univariate Cox regression analysis, all log-rank *p*-values of these five genes were <0.05 (Figure 3A). Following multivariate Cox regression analysis, the global *p*-value (log-rank test) of the five-gene prognostic model was only 0.000171 (Figure 3B). The AIC was 59.74, and the C-index was 0.89, indicating that these five genes may be favorable prognostic factors for the OS of patients with osteosarcoma. Moreover, the outcome of K–M survival analysis was consistent with that of the univariate Cox regression analysis (Figure 3C). Furthermore, *AMN1* and *ZP3* may be protective factors in osteosarcoma (HR: 1.26e−36 and 1.13e−07, respectively), whereas *LIMS1*, *SAMD4A*, and *SPARC* appear to be harmful factors in this setting (HR: 699.2, 167, and 298.7, respectively). Thus, a Lasso penalized Cox regression model including five SE-associated genes may be used to predict the OS of patients with osteosarcoma.

### Establishment and Validation of the Polygenic Risk Score Model

**FIGURE 4:**
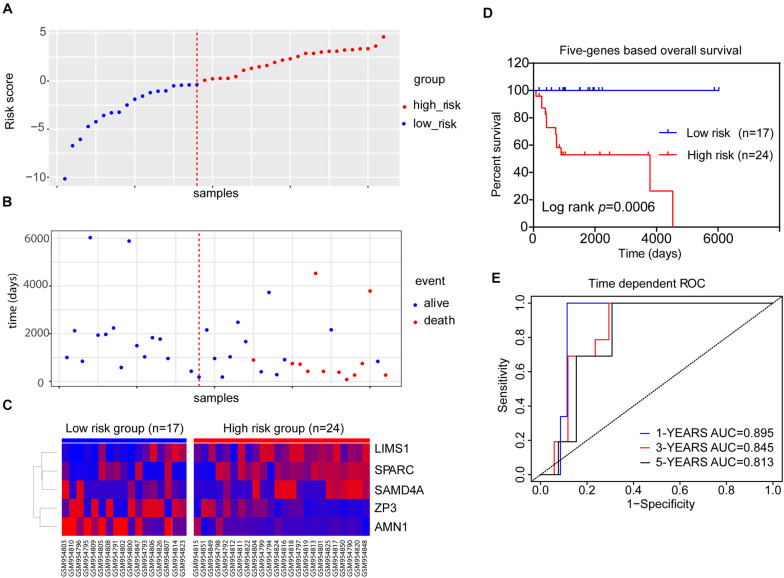
The five-gene risk score model for the GSE39058 dataset (*N* = 41). **(A)** Scatter plot displaying the risk score of each patient in the GSE39058 dataset. Patients were divided into high- or low-risk groups according to the risk score. The blue plots represent the patients in the low-risk group (risk score ≤ 0), while the red plots represent patients in the high-risk group (risk score > 0). **(B)** The survival status distribution of patients with osteosarcoma in the high- or low-risk groups. **(C)** The expression status of the five prognostic genes in 41 patients with osteosarcoma in the GSE39058 dataset. **(D)** K–M survival curves showing the difference in OS between high- and low-risk patients (41 patients) (log-rank test, *p* = 0.0006). **(E)** Time-dependent ROC curve analysis for the prediction of survival using the five-gene prognostic model. The AUCs of 1-, 3-, and 5-year OS are shown in the figure. AUC, area under the curve; K–M, Kaplan–Meier; OS, overall survival; ROC, receiver operating characteristic.

We integrated the expression data of the five genes and corresponding coefficient derived from the above multivariate regression analysis to establish the risk score model. All patients in the training dataset of GSE39058 (*N* = 41) were divided into high-risk (risk score > 0) and low-risk groups (risk score < 0) (Figure 4A). As shown in Figure 4B, survival was more commonly observed in the low-risk group, whereas death was more frequent in the high-risk group. *LIMS1*, *SAMD4A*, and *SPARC* tended to upregulated, whereas *AMN1* and *ZP3* were downregulated in patients of the high-risk group (Figure 4C). The K–M OS analysis predicted an inferior outcome of OS in the high-risk group (log-rank test, *p* = 0.0006) (Figure 4D). Meanwhile, the AUCs of a time-dependent ROC curve calculated using the five-gene-based risk score model were >0.8 (Figure 4E), indicating that the forecast model had high sensitivity and specificity.

The Target-OS dataset was further used to verify the predictive values of the polygenic risk score. Following the multivariate Cox regression analysis, the global *p*-value (log-rank test) of the five-gene prognostic model for the Target-OS dataset was <0.05 (Supplementary Figure S1A). A total of 88 patients were classified into the low- and high-risk groups using the optimal cutoff value of the risk score (Supplementary Figures S1B–D). The K–M curves of two groups based on risk scores were significantly different (log-rank test, *p* < 0.05), and AUCs of time-dependent ROC curves also showed that the five-gene risk model could be a favorable approach to predicting the OS of patients with osteosarcoma (Supplementary Figures S1E,F).

### Construction of the Predictive Nomogram Based on the Risk Score Model for Prognostic Prediction

**FIGURE 5:**
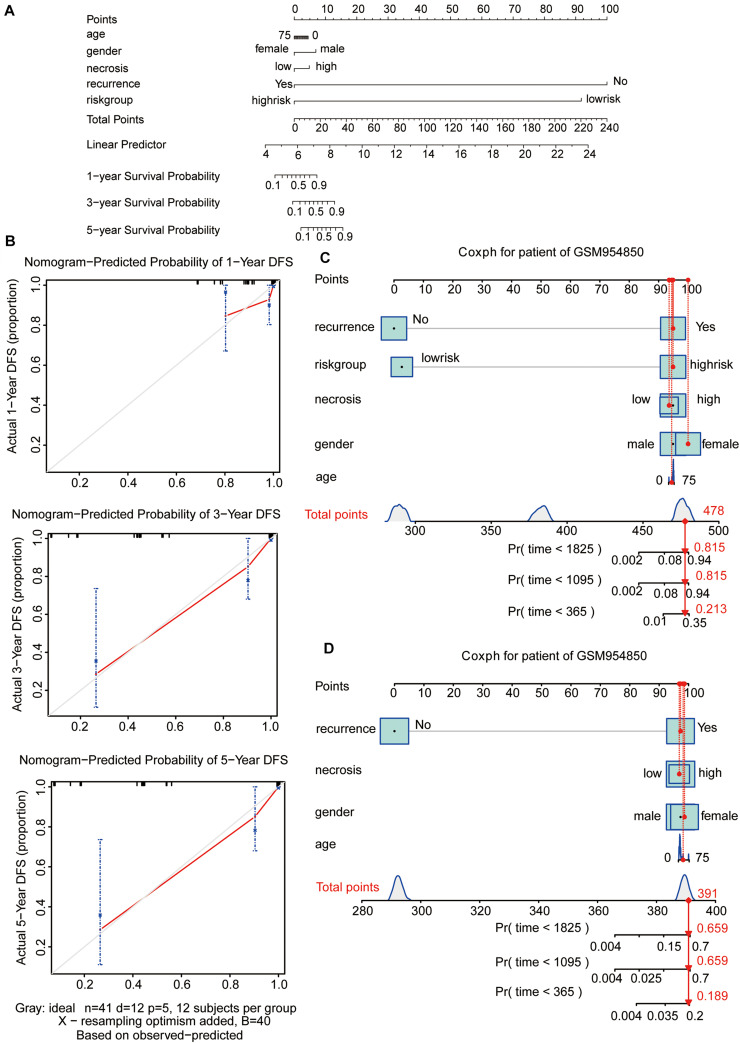
Nomogram predicting the probability of 1-, 3-, and 5-year OS in patients with osteosarcoma. **(A)** Nomogram adding up the points identified on the points scale (the upward line) for each variable. The total points projected on the bottom scales indicate the probability of 1-, 3-, and 5-year OS. **(B)** Calibration plot for predicting the 1-, 3-, and 5-year OS. The gray line represents the ideal condition. **(C**,**D)** The nomograms predicting the probability of 1-, 3-, and 5-year OS for the specific patient GSM954850 based on the model containing or not containing the risk group in the GSE39058 dataset. OS, overall survival; DFS, disease-free survival.

To visualize the Cox regression model, a nomogram was constructed for predicting the 1-, 3-, and 5-year OS probability for patients with osteosarcoma in the GSE39058 dataset (*N* = 41). The predictors of the nomogram included age, sex, necrosis, recurrence, and risk group (Figure 5A). The calibration plot shown in Figure 5B illustrates the performance of the nomogram. The calibration plots were close to the ideal prediction gray line (45° line), indicating that our nomogram performed well in predicting survival probability (Figure 5B). To assess the predictive effect of the risk group as an indicator of OS, we applied the nomogram to a specific patient (GSM954850) in GSE39058 (Figures 5C,D). The patient GSM954850 expired at 750 days. According to the model containing the risk group, the predicted probability of death at 1,095 days was 0.815; this value was markedly higher than that obtained for the model lacking the risk group (0.659). This finding implies that the predictive model containing the risk group as a parameter is more accurate than that lacking the risk group.

### Identification of SE-Related Hub Genes in Osteosarcoma Using WGCNA and a Protein–Protein Interaction Network

**FIGURE 6:**
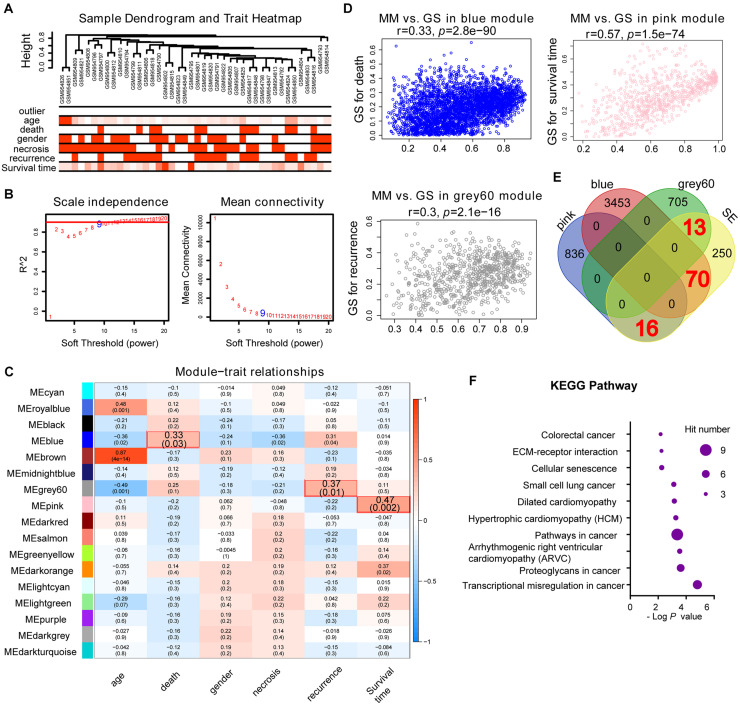
Identification of SE-related hub genes in osteosarcoma based on the GSE39058 dataset through WGCNA analysis. **(A)** Cluster analysis between the gene expression in the GSE39058 dataset and sample outliers or clinical traits. Color intensity was proportional to sample outliers, age, gender, necrosis, recurrence, and survival time. **(B)** Analysis of the scale independence and mean connectivity (vertical axis) for various soft-thresholding powers (β value of horizontal axis). **(C)** Heatmap of the correlation between modules and clinical traits of osteosarcoma; *p*-values in the table specify the correlation between modules and clinical traits. Three modules had high correlation with clinical traits: MEgray60 with recurrence (*r* = 0.37, *p* = 0.01); MEblue with death (*r* = 0.33, *p* = 0.03); and MEpink with survival time (*r* = 0.47, *p* = 0.002). **(D)** Scatterplot showing the MM versus GS of module genes related to death in the blue module, recurrence in the gray60 module, or survival time in the pink module (horizontal axis: MM means module membership, vertical axis: GS means gene significance). **(E)** Venn plot of SE-related genes and eigengenes in the blue, gray60, and pink modules. There are 70, 13, and 16 correlated genes, respectively. **(F)** KEGG pathway analysis of the above-correlated genes in the Venn plot. KEGG, Kyoto Encyclopedia of Genes and Genomes; SE, super-enhancer; WGCNA, weighted gene co-expression network analysis.

**FIGURE 7:**
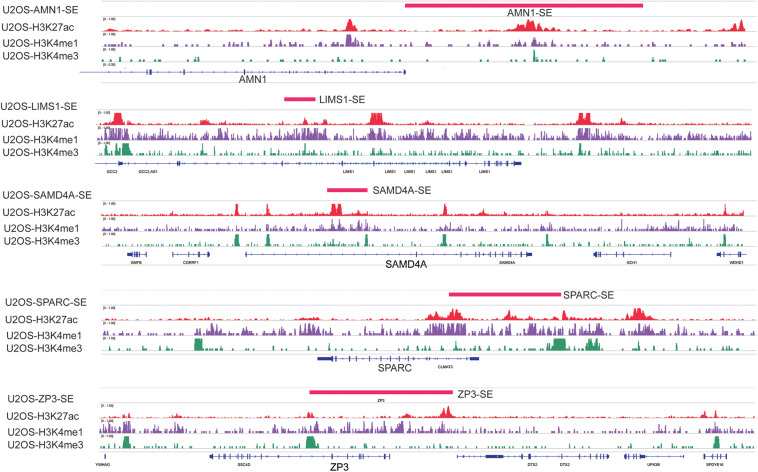
Signal tracks for H3K27ac (red), H3K4me1 (purple), and H3K4me3 (green) ChIP–seq profiles of the five-SE-associated hub genes visualized using IGV. The regions of SE are shown in a pink bar upon the signal tracks. ChIP–seq, chromatin immunoprecipitation–sequencing; SE, super-enhancer; IGV, Integrative Genomics Viewer.

Weighted gene co-expression network analysis is another bioinformatics method for the analysis of clinical traits linked to the levels of gene expression. Cluster analysis was performed to display the heatmap of clinical traits (Figure 6A). As shown in Figure 6B, β = 9 was selected as the best soft-thresholding value via prediction of the scale independence and mean connectivity (Figure 6B) to construct the gene co-expression network. Subsequently, eigengenes were divided into different colored modules, producing 17 different modules. A module–trait relationship heatmap was developed according to Pearson’s correlation coefficient (Figure 6C). Three modules presented higher correlation with clinical traits: MEgray60 with recurrence (*r* = 0.37, *p* = 0.01); MEblue with death (*r* = 0.33, *p* = 0.03); and MEpink with survival time (*r* = 0.47, *p* = 0.002) (Figure 6C). A scatter plot also showed that the MM of these three modules was positively correlated with GS (Figure 6D). A Venn plot of SE-associated genes derived from the SE-associated gene matrix was drawn, and the genes within three modules were intersected (Figure 6E). There were 13, 70, and 16 interaction genes between module gray60, blue, pink, and SE-associated genes. The interaction genes were enriched in numerous cancer-related pathways, such as proteoglycans in cancer and transcriptional misregulation in cancer (Figure 6F). In addition, we constructed a gene–drug interaction network using the five genes and related drugs (Supplementary Figure S2). All five genes interacted with JQ1 (an established SE inhibitor). The network depicted that these five genes were probably regulated by SE to a certain extent. Finally, we displayed the signal tracks for the H3K27ac, H3K4me1, and H3K4me3 ChIP–seq profiles of the five genes (Figure 7). We observed five predicted SEs near these five genes. These data suggest that the expression of the five genes is regulated by SE. In addition, the SE inhibitor JQ1 may regulate the expression pattern in U2OS cells.

## Discussion

Notably, there is a lack of effective target treatment regimens in clinical practice, except for the traditional method of surgical resection. Recently, prognostic models based on some disease-related genes or other biomarkers for improving the prognosis of osteosarcoma have attracted considerable attention. The Lasso regression Cox model, gene risk score model, and univariate and multivariate Cox regression models are widely used for the screening and mining of hub genes related to survival and other clinical characteristics. Particularly, the Lasso model can be applied to solve “curse of dimensionality” (small sample size combined with a very large number of genes) when large amounts of throughput biological data are processed. Recently, Lasso and WGCNA were used to construct a nomogram of radiomics of diagnostic computed tomography for predicting the survival of patients with high-grade osteosarcoma, which showed favorable performance (Wu et al., 2018). Another study also identified a seven-gene signature as a predictive biomarker for energy metabolism in osteosarcoma by Lasso and multivariate Cox regression (Zhu et al., 2020). Lasso coefficient profiles of the genes associated with the metastasis of osteosarcoma were established. These profiles were used to construct a risk score model for the prediction of metastasis of osteosarcoma (Dong et al., 2019). These findings imply that prognostic models based on different clinical traits and gene expression levels may help to potentiate the personalized treatment of patients with osteosarcoma.

In the present study, we constructed a five-SE-associated-gene-based nomogram prognostic model to improve the prognosis of patients with osteosarcoma. We first constructed an SE-associated gene expression matrix based on the 349 genes derived from the intersection of SE-associated genes and 20,819 genes from the GSE39058 dataset. According to this expression matrix, Lasso penalized Cox regression analysis was performed to screen a gene cluster containing *AMN1*, *LIMS1*, *SAMD4A*, *SPARC*, and *ZP3*, which was used to construct the risk score model based on the expression levels of five genes. Considering the group risk score as an independent indicator for prognosis, the K–M curve and log-rank test showed that a high risk score was correlated to augmented death ratio. We assessed the capability of the five-gene prognostic model in the GSE39058 dataset and also in the validation dataset Target-OS via univariate and multivariate Cox regression analyses and ROC curve analysis. The AUCs of the prognostic model for predicting the 1-, 3-, and 5-year OS were 0.895, 0.845, and 0.813, respectively, for the GSE39058 dataset and 0.549, 0.721, and 0.724, respectively, for the Target-OS dataset. The AUCs and C-index also showed that the five-gene prognostic model had a great performance for the prediction of survival and could be a robust and efficient model of prognosis. An interactive nomogram based on the risk score model and other clinical traits was constructed and identified using the calibration plots. To determine the predictive effect of the risk group as an indicator of OS, we also applied the nomogram to a specific patient (GSM954850) in the GSE39058 dataset. The predictive model containing the risk group as a parameter is more accurate compared with the nomogram model lacking the risk group. Through this approach, the prognosis of patients in the high-risk group may improve following the adjustment of individual treatment regimens for these patients.

The WGCNA of the 20,819 genes from the GSE39058 dataset revealed that the above five SE-associated genes also belonged to module genes. The KEGG analysis showed that the module genes associated with SE were enriched in numerous cancer-related pathways, such as proteoglycans in cancer and transcriptional misregulation in cancer. *LIMS1* functions as an oncogene to promote the survival of pancreatic cancer cells under oxygen–glucose deprivation conditions (Huang et al., 2019). The protein LIMS1 (also known as PINCH1) encoded by LIMS1 acts as an adaptor protein which contains five LIM domains or double zinc fingers (Hobert et al., 1999). LIMS1 can be involved in integrin signaling through interacting with integrin-linked kinase mediated by its LIM domain. Besides, LIMS1 can bind to integrin-linked kinase and parvin, which forms a protein complex, which is critical for the cell extracellular matrix adhesion (Ito et al., 2010). High expression of LIMS1 is associated with poor prognosis in human laryngeal carcinomas (Tsinias et al., 2018). Inhibition of LIMS1 through knockdown of LIMS1 can inhibit cell proliferation of neuroblastoma cells (Saeki et al., 2018). Thus, *LIMS1* may be considered as a biomarker for osteosarcoma. Secreted protein acidic and rich in cysteine (*SPARC*) encodes a cysteine-rich acidic matrix-associated protein, which is essential for extracellular matrix synthesis and epithelial–mesenchymal transition that promotes migration and invasion of many cancers (Sun et al., 2018; Jiang et al., 2019; López-Moncada et al., 2019). In particular, overexpression of *SPARC*, which was critical for the growth and maintenance of osteosarcoma, was found in 51 of 55 osteosarcoma tumor samples (Dalla-Torre et al., 2006). Overexpression of *SPARC* also mediated the suppressing effect of *TP53INP1* on the migration of pancreatic cancer cells and promoted the progression of malignant cancers (Seux et al., 2011). Therefore, it is convincing that *SPARC* can serve as a biomarker or treatment target for osteosarcoma. SAMD4A, encoded by the sterile alpha motif domain containing 4A (*SAMD4A*), was reported to involve in processes such as maternal RNA destabilization, translational repression, and early embryo development in the role of a posttranscriptional regulator (Smibert et al., 1996; Baez et al., 2011). Although studies about the function of *SAMD4A* in cancer are still rare, we believe that *SAMD4A* will become a novel biomarker for predicting the prognosis of osteosarcoma based on the findings of this study. Antagonist of the mitotic exit network 1 (*AMN1*) is essential for mitotic checkpoints and chromosome stability; the protein encoded by AMN1 is involved in the progression of cell cycle (Wang et al., 2003; Xie et al., 2019). Although there are no relative studies about the role of *ZP3* in osteosarcoma, even if in cancer, combined with the findings of our study, we should also pay attention to *AMN1* and *ZP3* in osteosarcoma in a certain extent. In the present study, the gene–drug interaction network of the five genes showed that all genes interacted with JQ1 (an established SE inhibitor). Moreover, according to the ChIP–seq signal of H3K27ac, H3K4me1, and H3K4me3, we detected strong signals of SEs around these five genes. These data support that the expression of the five genes may be regulated by SE, while JQ1 as an SE inhibitor probably regulated the expression pattern in U2OS cells. Further molecular mechanism studies are warranted to recover the relationship between expression and SE in the future.

In conclusion, this was the first study that used the Lasso model to screen prognostic indicators from the profile of SE-associated genes. The five-gene-based interactive nomogram may be used as a clinical tool for improving the prognosis of patients with osteosarcoma, offering novel insights into personalized therapy in this setting.

## Data Availability Statement

All datasets presented in this study are included in the article/Supplementary Material.

## Author Contributions

JQ and QL conceived and designed the theme of this study. ZO drafted the manuscript. TQ, GL, HZ, JW, and CT provided some helpful advice in several statistic methods. JQ and QQ revised the manuscript. The manuscript was revised critically by all authors involving in this study and all authors approved the final version with one accord.

## Conflict of Interest

The authors declare that the research was conducted in the absence of any commercial or financial relationships that could be construed as a potential conflict of interest.

## Footnotes

1 https://www.ncbi.nlm.nih.gov/geo/

2 https://cancergenome.nih.gov/

3 http://www.licpathway.net/sedb

4 http://cistrome.org/db/#/

## Supplementary Material

Supplementary Figure 1 Multivariate Cox regression analysis and risk score model analysis of the validation dataset Target-OS (*N* = 88). **(A)** Multivariate Cox regression analysis of dataset Target-OS based on the five-gene model. **(B)** Scatterplot displaying the risk score of each patient in the Target-OS dataset. The patients were divided into the high- or low-risk groups according to the risk score. The blue plots represent the patients in the low-risk group (risk score ≤ 0), while the red plots represent those in the high-risk group (risk score > 0). **(C)** Survival status distribution of the high- or low-risk groups in the Target-OS dataset. **(D)** The expression status of the five prognostic genes for patients in the Target-OS dataset. **(E)** K–M survival curves showing the difference in OS between high- and low-risk patients in the Target-OS dataset (log-rank test, *p* = 0.0438). **(F)** Time-dependent ROC curves analysis of survival prediction for patients in the Target-OS dataset using the five-gene prognostic model. The AUCs for 1-, 3-, and 5-year OS are shown in the figure. AUC, area under curve; K–M, Kaplan–Meier; ROC, receiver operating characteristic; Target-OS, Target-osteosarcoma, OS, overall survival; AIC, Akaike information criterion.

Supplementary Figure 2 Gene–drug interaction network analysis of the five genes and drugs.

## Abbreviations

AIC: Akaike information criterion
AUC: area under the ROC
C-index: concordance index
GEO: Gene Expression Omnibus
GS: gene significance
HR: hazard ratio
IGV: Integrative Genomics Viewer
K–M: Kaplan–Meier
Lasso: least absolute shrinkage and selection operator
MM: module membership
OS: overall survival
ROC: receiver operating characteristic
SE: super-enhancer
TCGA: The Cancer Genome Atlas
WGCNA: weighted gene co-expression network analysis.
