# Corrigendum: Construction of a Five-Super-Enhancer-Associated-Genes Prognostic Model for Osteosarcoma Patients

**DOI:** 10.3389/fcell.2020.631453

**Published:** 2021-01-19

**Authors:** Zhanbo Ouyang, Guohua Li, Haihong Zhu, Jiaojiao Wang, Tingting Qi, Qiang Qu, Chao Tu, Jian Qu, Qiong Lu

**Affiliations:** ^1^Department of Pharmacy, The Second Xiangya Hospital, Central South University; Institute of Clinical Pharmacy, Central South University, Changsha, China; ^2^Department of Pharmacy, Xiangya Hospital, Central South University, Changsha, China; ^3^Department of Orthopaedics, The Second Xiangya Hospital, Central South University, Changsha, China

**Keywords:** osteosarcoma, super-enhancer, prognostic model, Lasso, weighted gene co-expression network analysis, overall survival

In the published article, there were errors in the AUTHOR CONTRIBUTIONS section. Corrections have been made to **AUTHOR CONTRIBUTIONS**:

“JQ and QL conceived and designed the theme of this study. ZO drafted the manuscript. TQ, GL, HZ, JW, and CT provided some helpful advice in several statistic methods. JQ and QQ revised the manuscript. The manuscript was revised critically by all authors involving in this study and all authors approved the final version with one accord.”

The authors apologize for this error and state that this does not change the scientific conclusions of the article in any way. The original article has been updated.

