# Supplementary figures and images for: Construction of a Five-Super-Enhancer-Associated-Genes Prognostic Model for Osteosarcoma Patients
Source: Front Cell Dev Biol. 2020 Oct 30;8:598660. doi: 10.3389/fcell.2020.598660 (PMC7661850; doi:10.3389/fcell.2020.598660)

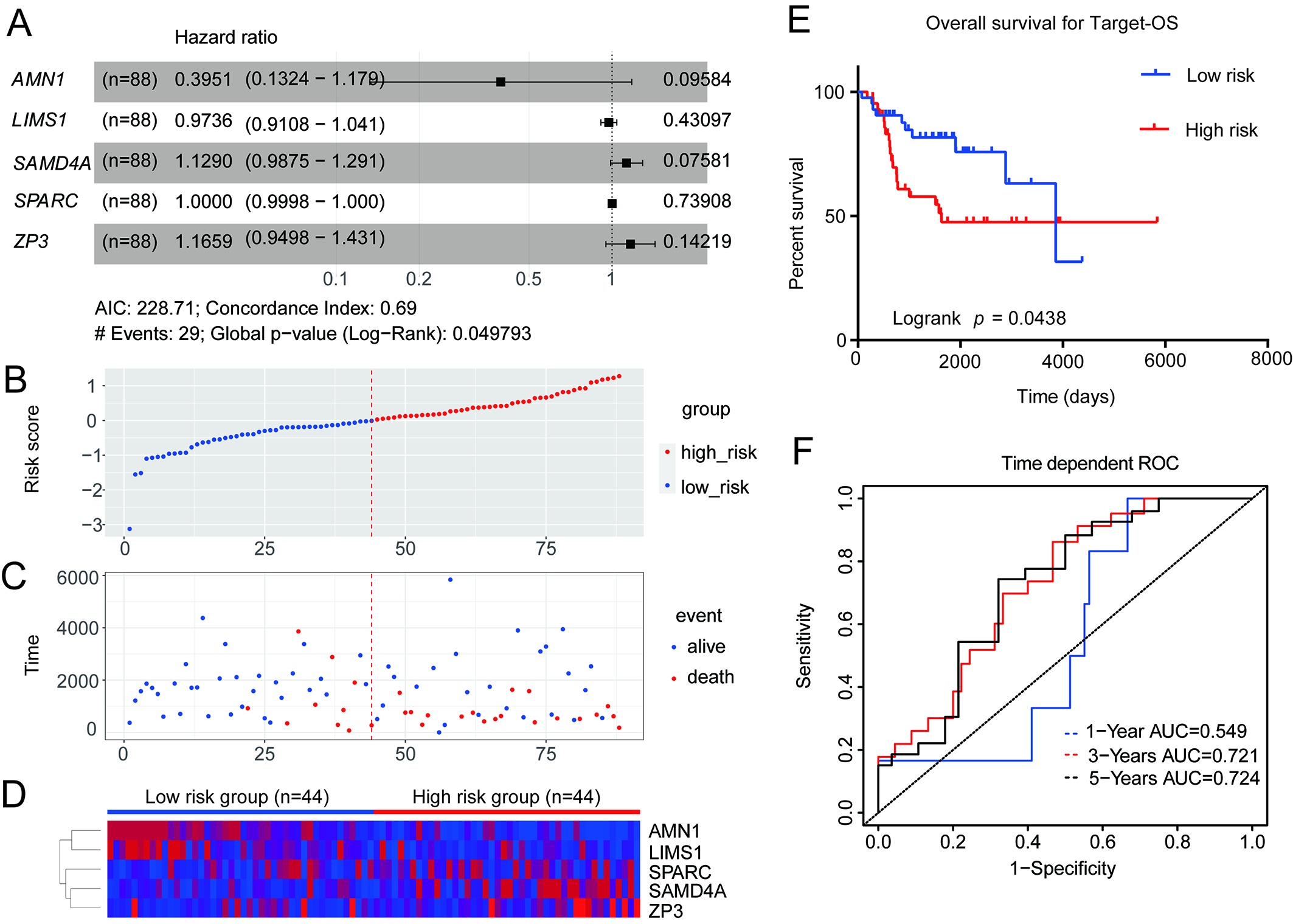

Supplement: Supplementary Figure 1 — Multivariate Cox regression analysis and risk score model analysis of the validation dataset Target-OS (N = 88). (A) Multivariate Cox regression analysis of dataset Target-OS based on the five-gene model. (B) Scatterplot displaying the risk score of each patient in the Target-OS dataset. The patients were divided into the high- or low-risk groups according to the risk score. The blue plots represent the patients in the low-risk group (risk score ≤ 0), while the red plots represent those in the high-risk group (risk score > 0). (C) Survival status distribution of the high- or low-risk groups in the Target-OS dataset. (D) The expression status of the five prognostic genes for patients in the Target-OS dataset. (E) K–M survival curves showing the difference in OS between high- and low-risk patients in the Target-OS dataset (log-rank test, p = 0.0438). (F) Time-dependent ROC curves analysis of survival prediction for patients in the Target-OS dataset using the five-gene prognostic model. The AUCs for 1-, 3-, and 5-year OS are shown in the figure. AUC, area under curve; K–M, Kaplan–Meier; ROC, receiver operating characteristic; Target-OS, Target-osteosarcoma, OS, overall survival; AIC, Akaike information criterion. [file Image_1.TIF]

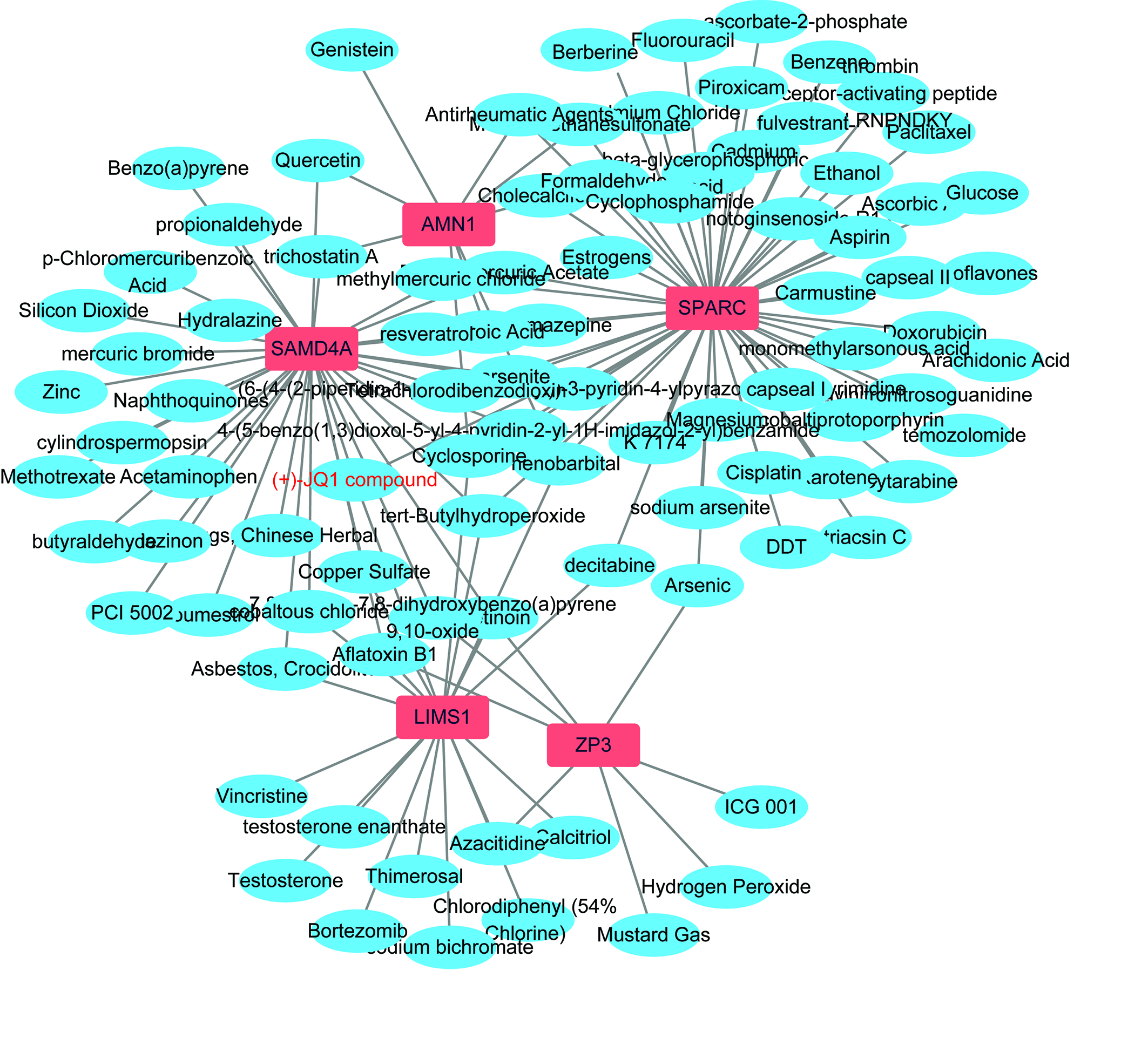

Supplement: Supplementary Figure 2 — Gene–drug interaction network analysis of the five genes and drugs. [file Image_2.TIF]
